# Towards real-time analysis of liquid jet alignment in serial femtosecond crystallography

**DOI:** 10.1107/S1600576722005891

**Published:** 2022-08-01

**Authors:** Jaydeep Patel, Adam Round, Johan Bielecki, Katerina Doerner, Henry Kirkwood, Romain Letrun, Joachim Schulz, Marcin Sikorski, Mohammad Vakili, Raphael de Wijn, Andrew Peele, Adrian P. Mancuso, Brian Ab­bey

**Affiliations:** a School of Computing, Engineering and Mathematical Sciences, La Trobe University, Melbourne, Victoria, Australia; b La Trobe Institute for Molecular Science (LIMS), La Trobe University, Melbourne, Victoria, Australia; c European XFEL, Schenefeld, Germany; d Australian Synchrotron, Australian Nuclear Science and Technology Organisation (ANSTO), Clayton, Victoria, Australia; Universität Hamburg, Germany

**Keywords:** machine vision, automation, liquid jet alignment, image processing

## Abstract

Liquid sample delivery systems are used extensively for serial femtosecond crystallography at X-ray free-electron lasers (XFELs). However, misalignment of the liquid jet and the XFEL beam leads to the X-rays either partially or completely missing the sample, resulting in sample wastage and a loss of experiment time. Implemented here is an algorithm to analyse optical images using machine vision to determine whether there is overlap of the X-ray beam and liquid jet. The long-term goal is to use the output from this algorithm to implement an automated feedback mechanism to maintain constant alignment of the X-ray beam and liquid jet. The key elements of this jet alignment algorithm are discussed and its performance is characterized by comparing the results with a manual analysis of the optical image data. The success rate of the algorithm for correctly identifying hits is quantified via a similarity metric, the Dice coefficient. In total four different nozzle designs were used in this study, yielding an overall Dice coefficient of 0.98.

## Abstract 2

A novel strategy is presented for sample jet alignment using machine vision for liquid-jet-based sample delivery systems. Feedback using height-resolution images from an optical microscope positioned perpendicular to the path of the X-ray beam enables tracking of the relative alignment of the liquid jet and X-ray beam.

## 1. Introduction

The sample delivery system is a key element of both serial femtosecond crystallography (SFX) and single-particle imaging experiments. The sample delivery rate should ideally be matched to the repetition rate of the X-ray free-electron laser (XFEL) source (Doppler *et al.*, 2022 ▸; Oberthuer *et al.*, 2017 ▸). This is to minimize sample consumption and also to minimize the amount of wasted beamtime by reducing the number of XFEL pulses where no sample is present (Grünbein *et al.*, 2018 ▸; Wiedorn *et al.*, 2018 ▸; Yefanov *et al.*, 2019 ▸). Maintaining a constant sample supply is challenging, as both systematic drifts and random fluctuations in the relative position of the X-ray beam and liquid jet can occur over time. The most commonly employed sample delivery system for SFX is the gas dynamic virtual nozzle (GDVN) (Gorel *et al.*, 2020 ▸; Sobolev *et al.*, 2020 ▸; Grünbein *et al.*, 2019 ▸; Vakili *et al.*, 2022 ▸; DePonte *et al.*, 2008 ▸); more recent versions of this injector include double-flow-focusing nozzles (DFFNs) (Oberthuer *et al.*, 2017 ▸; Vakili *et al.*, 2022 ▸) or incorporate rapid mixing (Hejazian *et al.*, 2021 ▸). The jet characteristics, such as its diameter, length and speed, can be controlled via the liquid and gas flow rates and the geometry of the nozzle(s) used. The jet diameter can be matched to the crystal size, which minimizes the background from the liquid and maximizes the probability that the X-ray beam intersects with the sample. The jet diameter is typically a few micrometres, which is comparable to the standard XFEL beam size (DePonte *et al.*, 2009 ▸). Because of the similar diameters of the liquid jet and X-ray beam, however, small misalignments in terms of their relative positions can have an impact on the data collection. It is critical, therefore, that these misalignments are continuously monitored and corrected for.

Misalignment between the liquid jet and X-ray beam can occur due to fluctuations in the liquid or gas flow rates, thermal variations between the various components involved in the liquid jet delivery and X-ray beam transport, or de­stabilization of the liquid jet due to the presence of crystals. These effects in turn introduce jitter and, potentially, drift in the liquid jet position. These instabilities, particularly drifts, become more significant factors the longer the experiment runs and can result in a decrease in data quality and quantity. Jet misalignment also represents a significant cost in terms of beamline resources and staff time required to monitor the degree of overlap between the X-ray beam and liquid jet.

The SPB/SFX instrument (Mancuso *et al.*, 2019 ▸) at the European XFEL (Tschentscher *et al.*, 2017 ▸) is one example of an XFEL instrument that performs serial crystallography routinely. A key goal at SPB/SFX is to implement a feedback loop for fine correction of the motor positions controlling the position of the nozzle in order to maintain optimum beam–jet alignment. The first step in achieving this is to develop an algorithm capable of accurately identifying whether or not the XFEL beam is intersecting the liquid jet. The development and testing of such an algorithm is the subject of the present article.

The underlying principle is to use machine vision to analyse optical microscopy images of liquid jets recorded at the instrument and determine the relative beam–jet position. Accurate determination of relative offsets is a crucial step for deciding if translation of the liquid jet needs to be performed to improve jet–beam overlap. We classify the optical data according to whether there is a ‘hit’ (when the X-ray beam intersects the jet) or a ‘miss’ (when it does not). We evaluate the performance of our algorithm by testing its assignment of hit or miss to consecutive pulses and comparing with data that have been classified manually. Being able to implement such an algorithm efficiently, and in real time, paves the way to automating the process of maintaining beam–jet alignment.

Machine learning (ML) was considered in the very first steps of the project. The reason why machine vision was ultimately selected over machine learning was because the relative simplicity of determining hits or misses from the optical data did not initially appear to warrant the development of a complex solution. Another reason for not opting to develop ML was that the ‘ground truth’ data sets against which the algorithm is benchmarked need to be classified manually and a significant amount of time is required to generate large numbers of classified frames. It was not clear at the time of first developing the algorithm whether a sufficiently large ‘training data set’ could be generated from a large enough variety of different types of nozzles to train an ML algorithm successfully. The machine vision approach, on the other hand, is a more conservative option that can reliably classify these hits and misses. As our confidence in the algorithm grows, ML may become another route to further performance improvement in practice.

## 2. Machine vision and experimental setup

**Figure 1:**
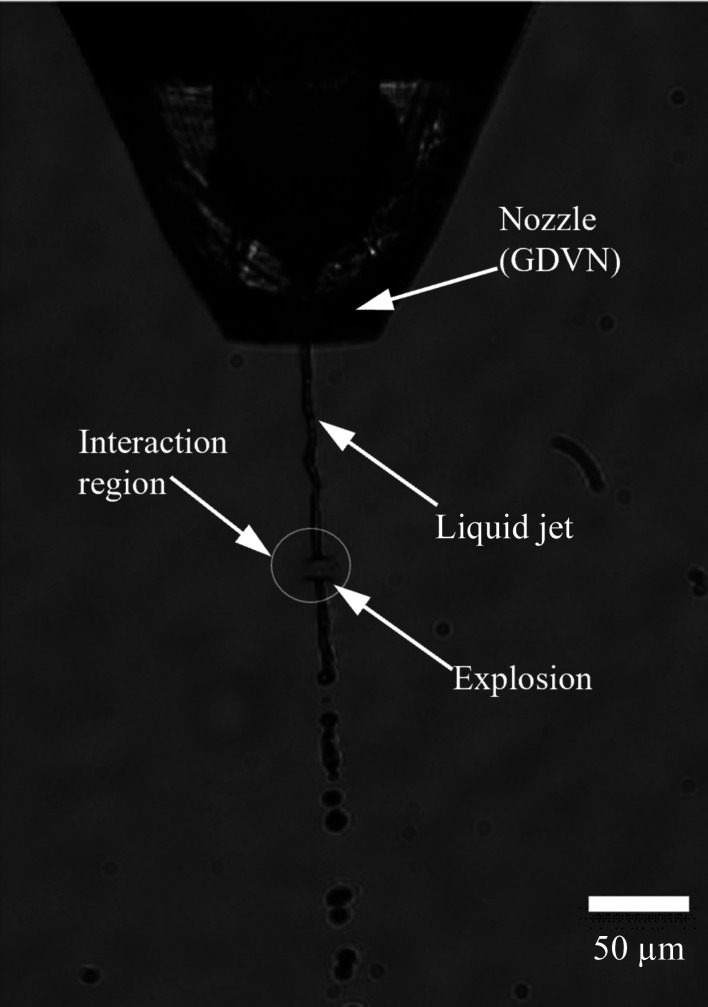
An optical microscopy image of a typical GDVN nozzle used for continuous delivery of crystals to the XFEL beam for SFX experiments. This image shows an example of a hit.

Machine vision has been widely employed for a range of research and industrial applications (Knoška *et al.*, 2020 ▸; Silva *et al.*, 2022 ▸). The flexibility, adaptability and optimization of machine vision make it an attractive option for providing real-time information about the relative liquid jet position based on optical microscopy data generated by the ‘side-view’ microscope which observes the liquid jet and is integrated into the SPB/SFX sample chamber (Schulz *et al.*, 2019 ▸). Fig. 1 ▸ presents a typical optical microscopy image generated by the side microscope. Key features of the image include the GDVN, the liquid jet which exits this nozzle, the jet explosion which occurs in the overlap region with the XFEL beam and the break-up of the jet (Wiedorn *et al.*, 2018 ▸; Pandey *et al.*, 2020 ▸; Stan *et al.*, 2016 ▸). The GDVN shown in Fig. 1 ▸ is a typical example of the types of sample delivery systems employed for SFX at the European XFEL (Silva *et al.*, 2022 ▸).

**Figure 2:**
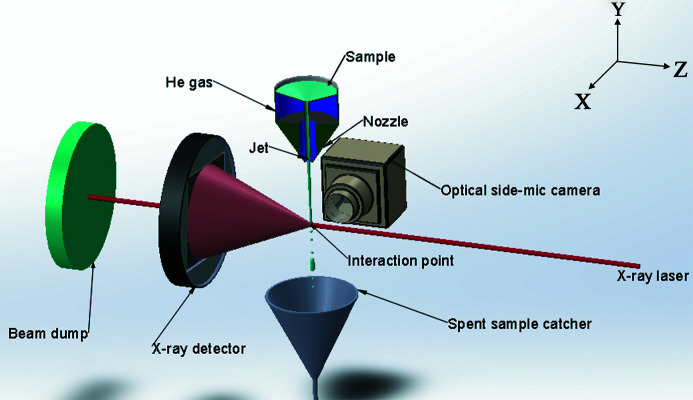
A schematic diagram of the setup in the vicinity of the sample interaction region on the SPB/SFX beamline. The optical side microscope camera (Oxford Instruments Andor Zyla 5.5sCMOS) is positioned perpendicularly with respect to the optical axis, 337 mm from the interaction region, and records images at a rate of 10 Hz to match the frequency of pulse trains arriving from the source. Not shown is the local vacuum enclosure around the nozzle.

The relative position of the side microscope with respect to the sample interaction region of the SPB/SFX instrument is shown in Fig. 2 ▸. The optical camera was set to record images at a rate of 10 Hz, significantly longer than the repetition rate of the source (1.1–4.5 MHz). In order to detect the break in the jet from a single pulse, the laser illumination of the jet is synchronized with the XFEL pulse train. The timing of the short pulsed laser (wavelength 532 nm, delay 880 ns) ensures that each optical data frame corresponds to a single XFEL pulse–jet interaction. In order to magnify the optical image on the camera, a lens with a numerical aperture of 0.28 was used.

## 3. Optical signature of XFEL–jet overlap

**Figure 3:**
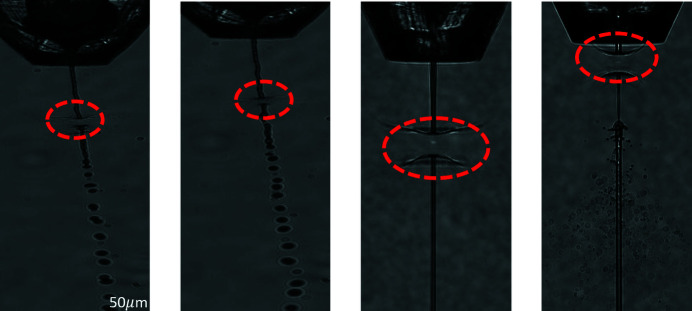
Four different examples of a hit in which the XFEL pulse overlaps with the liquid jet. The red ovals mark the interaction regions. As can be seen from the images, the break-up of the jet, the size of the jet explosion and the relative angle of the liquid jet with respect to the optical microscope camera can all vary significantly, complicating the automated analysis of the optical data.

The method employed here applies to SFX experiments where protein crystals suspended in a mother liquor are streamed within a continuous liquid jet to the interaction region of the XFEL beam (Darmanin *et al.*, 2016 ▸). The method of delivery is typically via the GDVN, although the algorithm developed here could apply and be adapted to any liquid jet. The XFEL beam is always incident normal to the liquid jet, and the optical camera is aligned to the normal of the plane of interaction of the jet and the XFEL pulse. Different examples depicting the interaction of the XFEL beam with the liquid jet (all hits) are shown in Fig. 3 ▸.

The alignment algorithm functions by scanning the optical images for signature(s) that indicate a jet explosion and a subsequent break in the jet. This interruption in the liquid flow is the key characteristic we wish to detect using machine vision and is used to classify the image as either a hit or a miss. This classification is an essential step in the implementation of an automated jet alignment algorithm. However, to be able to differentiate a hit from a miss reliably and reproducibly, pre-processing of the image data is required in order to account for noise in the image and variability in terms of the geometry of the liquid jet.

### 3.1. Template matching to determine region of interest

**Figure 4:**
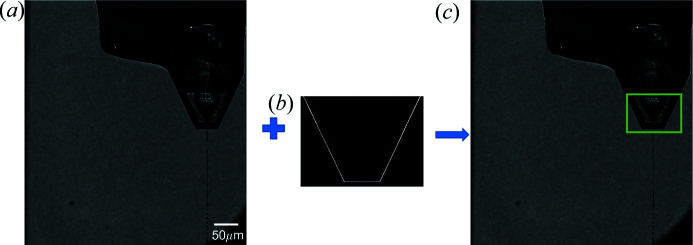
A schematic representation of template matching. (*a*) A target optical microscopy image. (*b*) A geometric template of the GDVN nozzle. (*c*) The nozzle is indicated by the green box identified via cross correlation, *i.e.* translating the template (kernel) across the target image shown in panel (*a*).

The first step in processing the optical images is known as ‘template matching’. Template matching is commonly employed in image analysis to locate a specific region of interest (ROI) within a larger image. A reference image or object is used as the ‘template’ which is then compared with the larger original image. The template is translated relative to the original target image. At each position of the template, the similarity of the template to that specific region of the image is assessed via a cross-correlation coefficient. Here, the template-matching step identifies the nozzle and thus defines the ROI for analysis on the basis of the cross-correlation maximum (Brunelli, 2008 ▸; OpenCV, 2020 ▸). In the present case the template is a simple geometric outline of the nozzle. The outline used was based on analysis of the images collected from the GDVN used in these experiments. For different nozzle designs it may be necessary to adapt the template or generate a library of different template designs. An example of template matching being implemented in order to determine the nozzle location is shown in Fig. 4 ▸.

**Figure 5:**
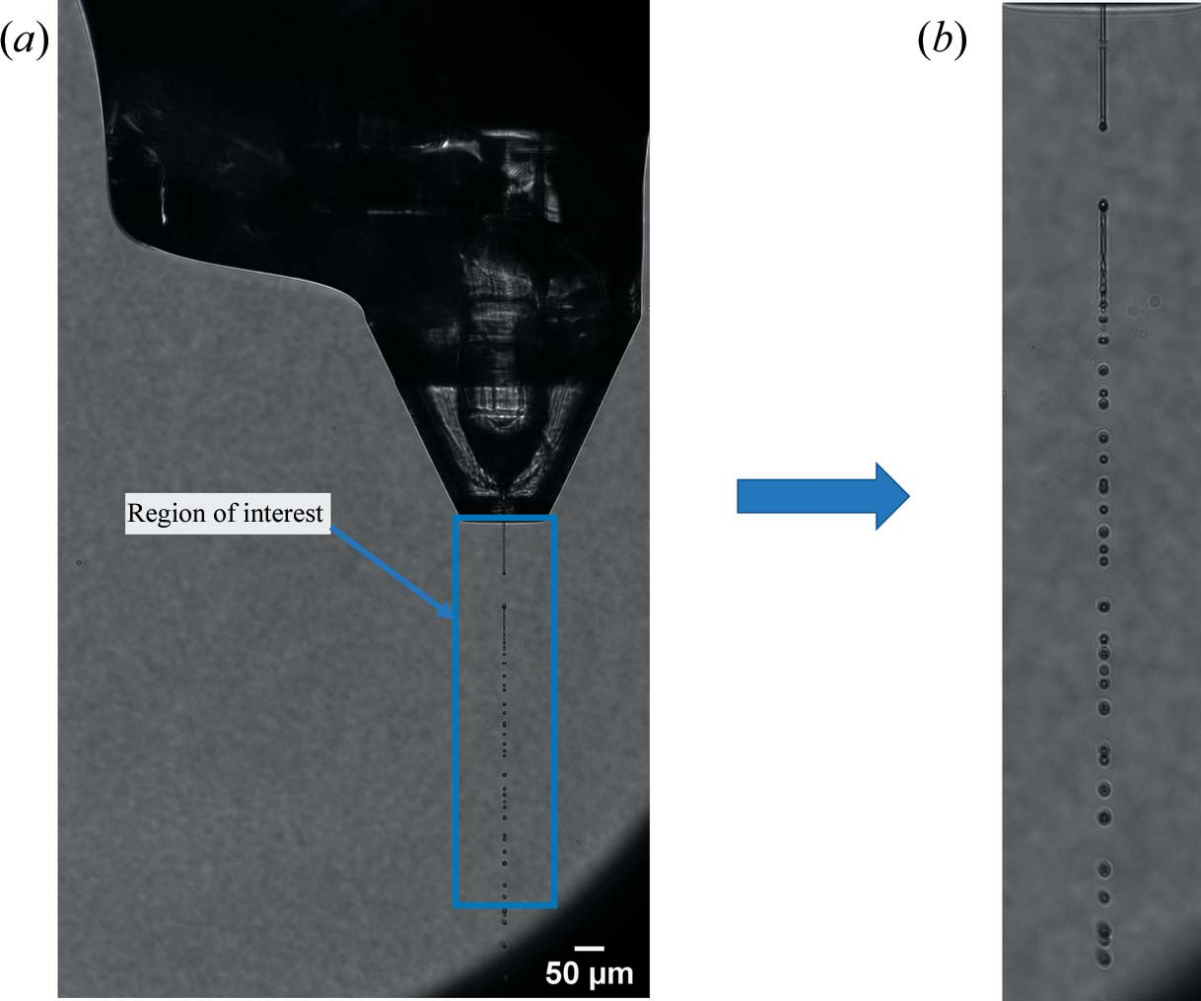
An example ROI from an optical microscopy image. (*a*) The blue rectangle shows the ROI which was automatically selected following the template-matching step to identify the nozzle. (*b*) The ROI is cropped out from the target image in preparation for further analysis.

Once the nozzle position has been identified from template matching it acts as a reference point in the image in order to determine the ROI for further analysis (Sahiner & Yagle, 1995 ▸). The motivation for defining an ROI was to reduce the computational overhead in terms of image analysis by reducing the field of view over which the analysis is performed. We note that currently the ROI is substantially larger than the break-up length of the jets examined in this paper. The motivation behind having such a large ROI was to be able to implement the same algorithm with a wide range of different nozzle parameters/jet lengths without requiring manual input from the user, as the location of the interaction point may vary from one experiment to another. An example ROI that has been defined following template matching is shown in Fig. 5 ▸. Following this step the ROI is cropped out from the larger image prior to further analysis being performed.

An analysis of thousands of optical microscopy images collected over the period of a standard experiment indicated that the background and nozzle features in the image remained reasonably constant, whilst significant changes in the character and position of the liquid jet were observed. Once the ROI was identified it was cropped from the image and passed to the next step to determine if a jet explosion had occurred. The first step in analysing the ROI was binarization of the greyscale image.

### 3.2. Binarization and thresholding

**Figure 6:**
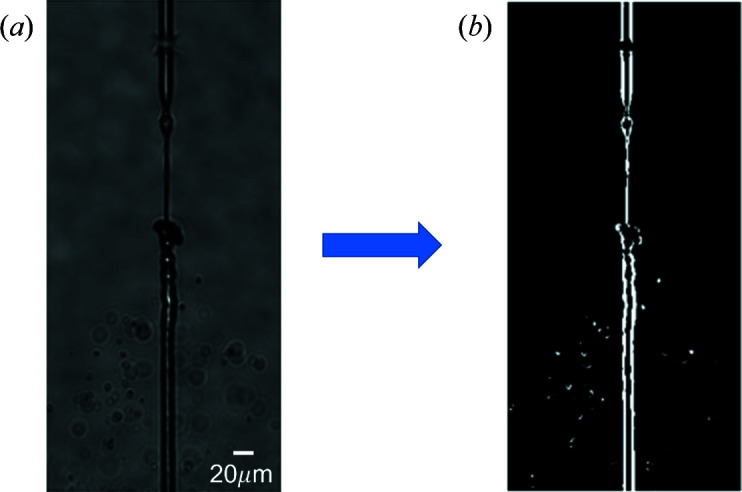
Conversion of a greyscale optical microscopy image to a binary image. (*a*) The original 16-bit greyscale image. (*b*) The same image after binarization.

Converting a greyscale image to a binary one allows us to simplify the analysis and extract the key features which provide a signature for jet explosion. The process of binarization converts each of the greyscale image pixels to either a zero or a one, depending on a set threshold value. To account for any non-uniformities in the illumination, the threshold value at each pixel was calculated using a sum of the Gaussian-weighted average minus a user-defined offset value of *C* = 7 within a window of 13 × 13 pixels (*W* = 13) centred on the target pixel (Bataineh *et al.*, 2011 ▸). These settings worked well for the range of nozzles and conditions tested here, but may need to be adjusted to achieve optimal performance of the algorithm for individual experimental conditions (Otsu, 1979 ▸; Roy *et al.*, 2014 ▸; Eggers & Villermaux, 2008 ▸). The choice of ROI also affects the effective signal-to-noise ratio (Jiang *et al.*, 2007 ▸) for ‘measuring’ the jet explosion as it limits the analysis to a region likely to contain the interaction between the XFEL beam and liquid jet. Limiting the ROI also helps to make the algorithm more robust to any variations in the illumination uniformity across the image (Finlayson, 2018 ▸; Zhang & Li, 2015 ▸). In the present case we have processed 16-bit images and thus had relatively fine control over the threshold value relative to the total range. The automation and selection of the threshold value for binarization was a key step in the successful implementation of the jet alignment algorithm (techtutorialsx, 2019 ▸). An example of binarization of the liquid jet is presented in Fig. 6 ▸. After the binarization step, the processed image is ready for analysis to identify any potential break in the jet, indicating an explosion has occurred which is characteristic of a hit.

### 3.3. Scanning for jet explosion

**Figure 7:**
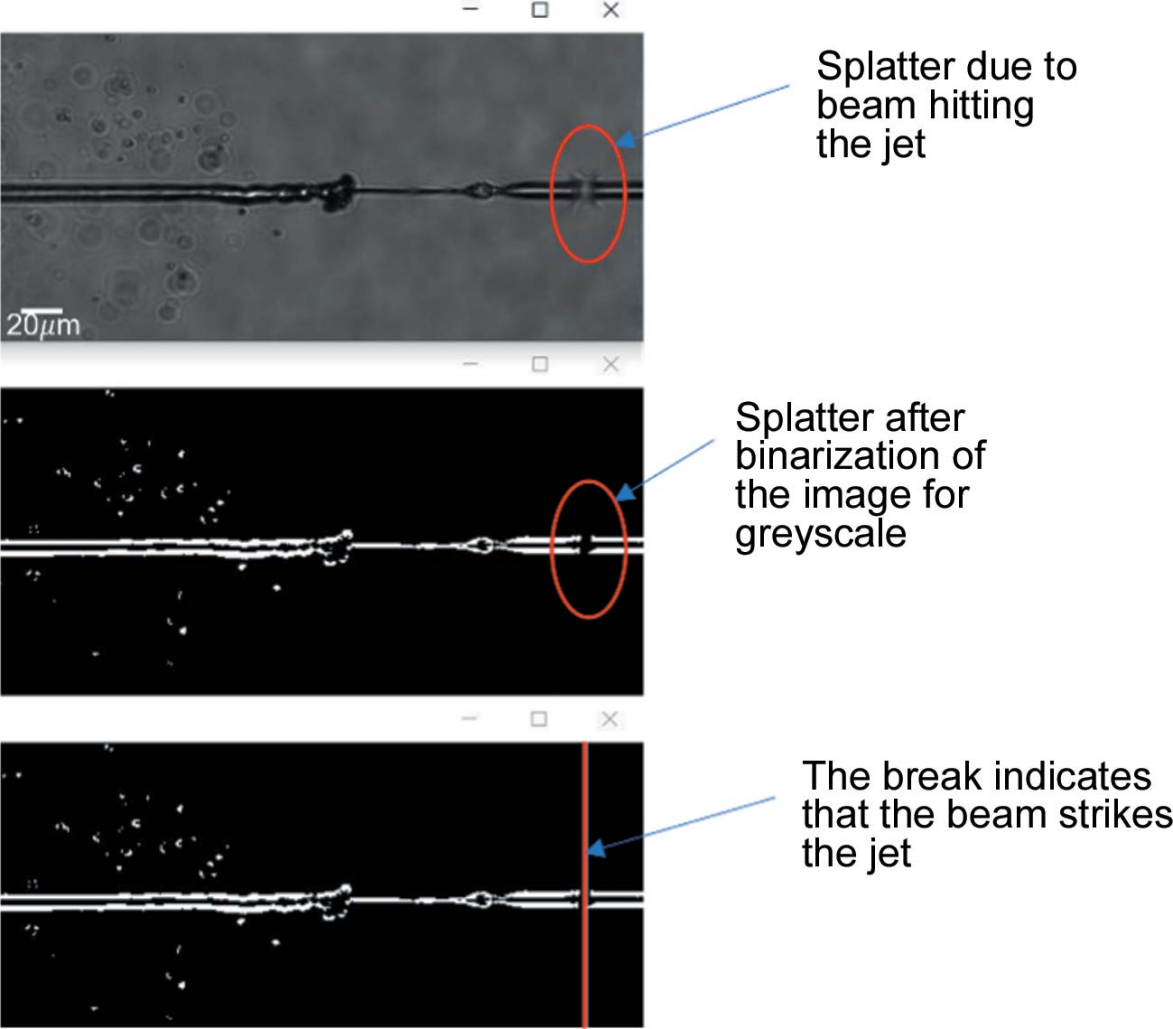
Illustration of the process of locating a break in the liquid jet following binarization of the cropped ROI. (Output window of the algorithm.)

Jet explosion was characterized across all of the primary liquid sample delivery systems used at the European XFEL, including the GDVN and DFFN (Eggers & Villermaux, 2008 ▸). Even though the jetting parameters, such as the nozzle diameter or flow rate, vary depending on the mother liquid and sample crystal being used, each jet explosion caused by a hit from the XFEL beam still displays similar image characteristics and so can be analysed using the same algorithm. As such, the utility of this method is maintained across different liquid jet delivery systems. Where there is a dis­continuity in the binarized jet at the interaction point this is always classed as a hit. Discontinuities can also occur at the point where the liquid stream breaks up into droplets (*e.g.* see Fig. 5 ▸) but these typically form more than 200 µm from the interaction region and can be readily excluded by applying a simple test of distance from the nozzle. Accordingly, a hit is defined by the algorithm as the presence of a simultaneous discontinuity on both sides of the binarized jet that is within 200 µm of the nozzle. Detection of discontinuities or breaks in the jet is accomplished by performing a row-by-row (or equivalently a column-by-column) scan of the binarized image. In the image, pixels within the jet are assigned a value of 1 (white pixels) and the background pixels are assigned a value of 0 (black pixels). If a continuous row (or column) of white pixels is detected, followed by a grouping of black pixels (up to a width of 20 pixels), and then another continuous row (or column) of white pixels, the region is identified as being a break in the jet. An example of this is shown in Fig. 7 ▸.

Other factors which needed to be considered when classifying an image as either a hit or a miss were the frame rate of the camera and the number of consecutive images that need to be considered before reliably determining whether the beam and jet are misaligned.

### 3.4. SMA classifier for hits or misses

Imaging noise and short-timescale (<100 ms) jet variations can cause the algorithm to record a false positive, identifying a hit when there has been no jet–beam interaction, or a false negative, assigning a miss when there has in fact been a hit. The optical camera recorded images at a rate of 10 Hz, corresponding to the X-ray pulse train frequency of the XFEL. Given that systematic jet drift and correction typically occur on timescales much longer than 100 ms, it was possible to gain a significant improvement in the reliability of the jet alignment algorithm by employing a simple moving average (SMA). In the present work, an SMA utilizing a sliding window width comprising ten consecutive images was used. If one of the ten images was registered as a hit then the entire 1 s ‘instance’ of measurement time was registered as a hit. For each new frame of data (image), the SMA sliding-window function works by discarding the oldest frame of data, causing the last location in the buffer to empty, and adding the newest image generated by the optical microscope. This type of SMA implementation is often referred to as a ‘first-in-first-out’ or ‘FIFO’ buffer arrangement (Dan & Towsley, 1990 ▸; Kartheek & Nageswararao, 2020 ▸). The buffering time for the camera is 100 ms. It should be noted, therefore, that to operate in ‘real time’ the algorithm should be able to process and classify the image data in, ideally, less than 50 ms to account for the frame rate/buffering time without introducing lag into the system. The reliance on a moving-average analysis to increase the reliability of the algorithm also has implications for the response time. For example, it would currently take around 0.5 s (equivalent to multiple pulse trains arriving) for the algorithm to register a hit or a miss and consequently trigger a motor movement or other corrective action. Hence, practically, ‘real time’ in the present context means that the algorithm is able to provide an output on a timescale of <1 s rather than on a pulse-to-pulse basis, which is sufficient for the scale of drifts observed in experiments to date.

### 3.5. Summary of the algorithm

**Figure 8:**
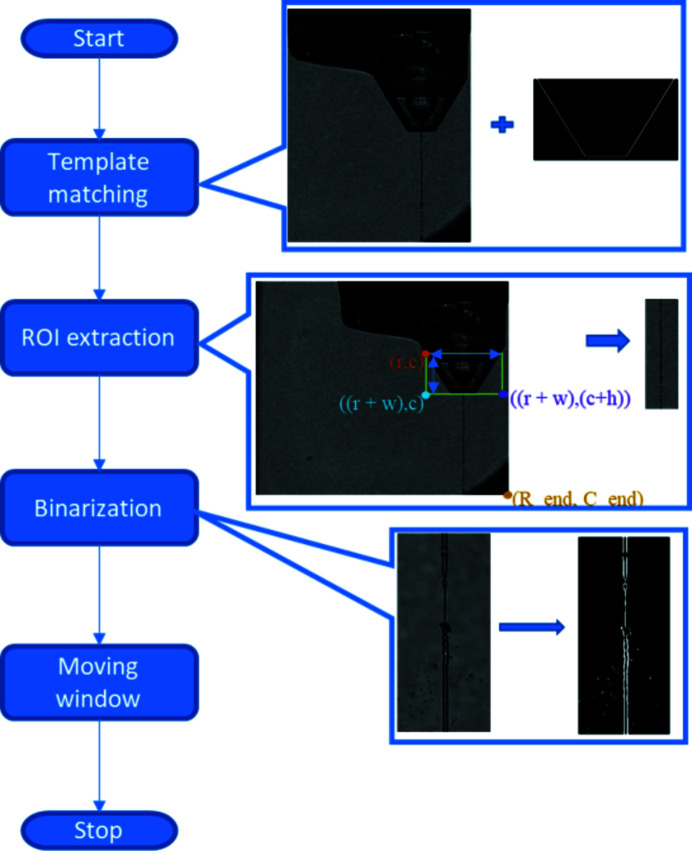
(Left) A flow diagram providing an overview of the basic processes in the jet alignment algorithm. (Right) Some example images representing the key steps.

Combining template matching, ROI cropping and binarization using an adaptive threshold, followed by a FIFO approach based on an SMA, enables classification of the optical microscopy images as either a hit or a miss. Fig. 8 ▸ presents a flow chart providing an overall summary of the key steps in the algorithm.

For the current implementation of the algorithm, the Python programming language was used. The desktop PC running the algorithm used a standard Windows 10 OS with an i5 processor running at 1.7 GHz and 16 GB of RAM. The algorithm could typically process around 20 frames of optical data per second on this system.

## 4. Discussion of results from applying the algorithm

In order to provide experimental validation of the jet hit or miss algorithm, we used optical microscopy data collected during a jet characterization experiment performed at the European XFEL (Vakili *et al.*, 2022 ▸). For the initial tests of the algorithm’s performance, we employed pure water jets that did not contain any crystalline sample. This enabled refinement and optimization of the algorithm parameters to be carried out prior to application to real-world sample data. There were four nozzles used to create this data set with four different aperture geometries, and three different liquid and gas flow rates were used per nozzle type to achieve different liquid jet diameters. The carrier liquid used for all nozzles was identical (*i.e.* water) and thus the liquid viscosity was the same for all four nozzles.

A quantitative analysis of the algorithm’s performance was carried out and the Dice coefficients calculated (Gaillard, 2005 ▸). For a given sequence of images, the Dice coefficient is calculated by comparing the algorithm output with a comparative data set of ‘correctly’ classified hits and misses generated by manual assessment of each of the optical images. This assessment was performed post-experiment without any time constraints and can be readily performed by a human. The procedure is identical to the one which is normally carried out manually by beamline staff during the experiment to determine if there is overlap of the XFEL beam and liquid jet. Assuming that the manual classification scheme is 100% accurate, which is a reasonable assumption given enough time, this enabled the determination of a figure of merit for the performance of our algorithm. Depending on whether the result from the algorithm matched the comparative data set, an instance was classified as true positive (TP), true negative (TN), false positive (FP) or false negative (FN). These values define the Dice coefficient as 2TP/(2TP + FP + FN), and accordingly a Dice coefficient of 1 represents complete agreement between the sets and a Dice coefficient of 0 represents no correctly classified events.

### 4.1. Algorithm fidelity check

A false positive is when the algorithm determines that there has been a hit when in fact there has been a miss; conversely, a false negative occurs when the algorithm incorrectly identifies a hit as a miss. Although the overall performance of the algorithm for the data sets tested here is very promising, there is still some room for improvement. For example, with the XFEL beam on, the percentages of FPs and FNs were 1.9 and 2.7%, respectively (see Table S1 in the supporting information). An analysis of individual images associated with FPs and FNs reveals that, in the majority of cases, these were due to either ‘partial hits’ or an unstable liquid jet. Partial hits are where the X-ray beam intersects the edge of the liquid jet rather than the centre. In general, any sign of interaction between the jet and the X-ray beam was manually classified as a hit, yet the majority of these partial hits were incorrectly classified by the algorithm as misses (FNs). In the case of a temporarily unstable jet, the early formation of droplets and greatly reduced break-up length caused the algorithm to determine incorrectly that the jet was broken and thus a hit had occurred (FP). Since it is useful for the operator to know when a partial hit or unstable jet has occurred, a further improvement to the algorithm could potentially be to introduce additional image classification for these two cases. This would involve looking for features specific to partial hits (*e.g.* ‘necking’ or a sudden reduction in jet diameter) or specific to an unstable jet (*e.g.* rapid movement of the jet angle between frames). These improvements to the algorithm will be explored as part of our future work.

**Figure 9:**
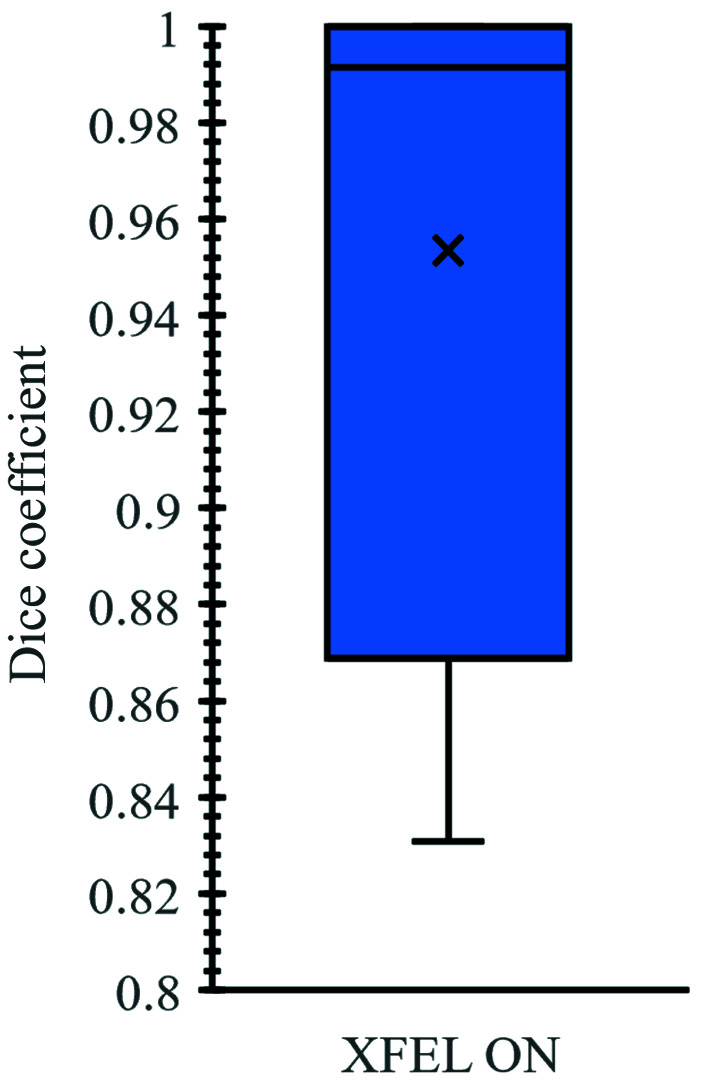
A summary of Dice coefficients for the case where the XFEL is on (*N* = 39 000 images), shown on the left axis. The ‘×’ denotes the mean, the horizontal line denotes the median, and the box indicates the lower quartile, the median and the upper quartile. The whiskers indicate, respectively, the smallest and largest observations.

Data sets at the European XFEL are divided into ‘runs’. These runs consist of a sequential series of measurements, which comprise between 1000 and 2500 individual frames, and which typically correspond to near-identical experimental conditions. The box-and-whisker plot in Fig. 9 ▸ summarizes the results of the Dice coefficient results for each of the 39 runs (*N* = 39 000 images) analysed when the XFEL beam was on. With the XFEL beam on, the algorithm achieves Dice coefficients ranging from 0.83 to 1. The mean value for the Dice coefficient is 0.98, with the standard deviation σ = 0.04 (Vakili *et al.*, 2022 ▸; Knoška *et al.*, 2020 ▸).

**Figure 10:**
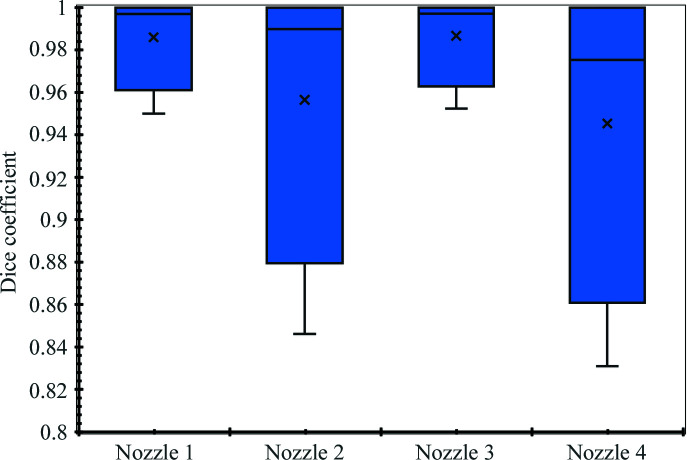
Box-and-whisker plots summarizing the performance of the hit and miss classification algorithm for the different nozzles that were used during the experiment. The nozzle characteristsics are as follows: Nozzle 1 = MVED_B (*N* = 9600 images), Nozzle 2 = JKMH#5 (*N* = 13 800 images), Nozzle 3 = JKMH#6 (*N* = 8400 images) and Nozzle 4 = MVED_C (*N* = 7200 images). Design files and details regarding the device geometries can be accessed at https://github.com/flmiot/EuXFEL-designs. The ‘×’ symbols denote the mean, the horizontal lines denote the median, and the boxes indicate the lower quartile, the median and the upper quartile. The whiskers indicate, respectively, the smallest and largest observations.

Four different types of nozzle were used to generate the ‘XFEL on’ data. We calculated separate Dice coefficients for the performance of each of the four nozzles to see whether different nozzles generated data sets that could be more reliably classified, and the results are summarized in Fig. 10 ▸. We did this both to test the robustness of the algorithm and to determine whether certain nozzles maintained a more reliable alignment, which could potentially help to inform the choice of nozzles for future experiments. From Fig. 10 ▸ it does appear that there are variations in the overall performance of the algorithm for the different nozzles, although based on the data analysed these differences may not be statistically significant. Nozzles 1 and 3 each have smaller data ranges and higher mean Dice coefficients (noting that similar numbers of images contributed to each data set) than the others, suggesting that certain nozzles may produce more reliable classification when analysed using our algorithm. However, for all nozzles the algorithm produced a mean Dice coefficient above 0.9, demonstrating that the algorithm will be ‘accurate’ more than 90% of the time for all nozzles tested.

We note that another factor contributing to algorithm accuracy may be the fact that thicker liquid jets produce a substantially larger amount of splatter when hit by the XFEL pulse. Since the algorithm can more reliably detect the interaction of the XFEL beam with the liquid jet when there is a clear splatter pattern in the image, the size of the splatter pattern is inversely proportional to the probability of occurrence of, for example, FNs. In addition, due to high variance in the image pixel intensities for thicker liquid jets, the resulting binarized image will have sharper and more well defined edges, which is a critical requirement for any vision-based system (Syed *et al.*, 2020 ▸).

In addition to the algorithm being unable specifically to distinguish partial hits and the presence of an unstable jet, there are other limitations which could potentially impact the algorithm’s performance. One of these is the current ROI size, which could be reduced to match the specific characteristic break-up length of the jet being monitored. This could be achieved either through user input, or via an additional automated step at the start of the algorithm to select only the area in the local vicinity of the interaction region for analysis. Another potential limitation is the fact that optical images are processed and analysed sequentially. Classification currently takes approximately 50 ms on a standard desktop computer, which is less than the rate at which optical images are collected. However, if the optical camera frame rate were ever increased, or if a more complex and computationally intensive image analysis was required, the implementation of the algorithm could be parallelized, enabling multiple images to be processed at the same time.

## 5. Conclusions and future work

The jet hit and miss classification algorithm discussed here shows promise as a way of monitoring the overlap of the XFEL beam and the liquid jet in real time. Following the steps outlined in this paper, we have demonstrated a mean Dice coefficient performance of 0.95 for liquid jets with the X-ray beam on. Furthermore, no false positives were recorded with the beam off.

The algorithm can be used to investigate the performance of different delivery systems and would allow the investigation of the effect of factors such as choice of sample carrier liquid, sample size and type, liquid and gas pressure, nozzle type and geometry, and gas choice. A key focus of the next stage in development will be to test the algorithm under a wider range of different conditions and develop a feedback system where a miss determined by the algorithm can be coupled with image detection of the jet position to translate into motor movements that will automatically re-align the jet and the X-ray beam. It is envisioned that incorporating this program into the existing control apparatus which controls the movement of the nozzle and other sample delivery mechanisms on the SPB/SFX instrument will increase measurement efficiency, save instrument staff time, reduce sample wastage and, ultimately, lead to larger volumes of SFX data being collected.

The next phase of our work will involve implementation of the algorithm on the SPB/SFX instrument to provide *in situ* feedback regarding the (mis-)alignment of the liquid jet. Initially, this will involve any miss triggering an alarm which will alert the user to the need to re-align the jet. In the longer term, the output of the algorithm will be used to develop an automated correction to the jet parameters. However, note that the liquid jet overlap with the XFEL beam depends not only on the nozzle position but also on the flow rate and sheath gas pressure. The interplay of these jet parameters will be explored as a function of the output of the machine-vision-based jet alignment algorithm.

## Supplementary Material

Additional table summarizing results. DOI: 10.1107/S1600576722005891/ap5045sup1.pdf
