# Supplementary material for: Towards real-time analysis of liquid jet alignment in serial femtosecond crystallography
Source: J Appl Crystallogr. 2022 Aug 1;55(Pt 4):944–52. doi: 10.1107/S1600576722005891 (PMC9348884; doi:10.1107/S1600576722005891)
Supplement: Supplementary file 1 [file j-55-00944-sup1.pdf]

JOURNAL OF
APPLIED
CRYSTALLOGRAPHY

Volume 55 (2022)

Supporting information for article:

Towards real-time analysis of liquid jet alignment in serial femtosecond crystallography

Jaydeep Patel, Adam Round, Johann Belieki, Katerina Doerner, Henry Kirkwood, Romain Letrun, Joachim Schultz, Marcin Sikorski, Mohammad Vakili, Raphael de Wijn, Andrew Peele, Adrian P. Mancuso and Brian Abbey

Table S1 Summary of the results for True Positive (TP), True Negative (TN), False Positive (FP), and False Negative (FN) as well as the corresponding Precision ($\boldsymbol{TP/(TP+FP)}$), Recall ($\boldsymbol{TP/(TP+FN)}$), and F1 scores ($\boldsymbol{TP/(TP+0.5\cdot(FP+FN))}$) for all of the data sets analysed with the XFEL beam on.

Run	TN	FN	FP	TP	Precision	Recall	F1	Dice coefficient
run5	294	13	14	288	0.95	0.96	0.96	0.96
run6	248	54	10	291	0.97	0.84	0.90	0.90
run8	314	15	15	292	0.95	0.95	0.95	0.95
run10	162	10	19	407	0.96	0.98	0.97	0.97
run12	0	15	18	570	0.97	0.97	0.97	0.97
run14	212	12	13	364	0.97	0.97	0.97	0.97
run16	0	15	78	531	0.87	0.97	0.92	0.92
run18	50	5	60	484	0.89	0.99	0.94	0.93
run20	15	42	21	523	0.96	0.93	0.94	0.95
run22	33	39	35	497	0.93	0.93	0.93	0.93
run38	20	33	55	526	0.91	0.94	0.92	0.92
run40	52	2	1	573	1.00	1.00	1.00	1.00
run42	43	4	1	600	1.00	0.99	1.00	1.00
run44	524	19	9	98	0.92	0.84	0.88	0.88
run46	0	15	118	470	0.80	0.97	0.88	0.89
run48	0	15	83	505	0.86	0.97	0.91	0.92
run50	45	55	34	500	0.94	0.90	0.92	0.92
run52	122	6	3	513	0.99	0.99	0.99	0.99
run54	30	6	61	555	0.90	0.99	0.94	0.94
run70	0	5	8	590	0.99	0.99	0.99	0.99
run72	0	19	31	608	0.95	0.97	0.96	0.98
run74	0	31	61	559	0.90	0.95	0.92	0.90

**run76**	0	45	48	510	0.91	0.92	0.92	0.91
**run78**	0	20	65	552	0.89	0.97	0.93	0.93
**run95**	0	32	56	537	0.91	0.94	0.92	0.93
**run97**	5	41	29	562	0.95	0.93	0.94	0.95
**run99**	10	65	20	563	0.97	0.90	0.93	0.94
**run101**	32	45	78	489	0.86	0.92	0.89	0.87
**run103**	15	61	21	551	0.96	0.90	0.93	0.92

We note that in the case that the XFEL beam was off, the TP values and hence Dice coefficients are necessarily zero in every case. The percentage of FP and FN with the XFEL beam off was found to be negligible (both were < 0.01%).
